# Gamma-glutamyl cycle in plants: a bridge connecting the environment to the plant cell?

**DOI:** 10.3389/fpls.2015.00252

**Published:** 2015-04-16

**Authors:** Antonio Masi, Anna R. Trentin, Ganesh K. Agrawal, Randeep Rakwal

**Affiliations:** ^1^Dipartimento di Agronomia Animali Alimenti Risorse Naturali e Ambiente (DAFNAE), University of PadovaLegnaro, Italy; ^2^Research Laboratory for Biotechnology and BiochemistryKathmandu, Nepal; ^3^GRADE (Global Research Arch for Developing Education) Academy Private LimitedBirgunj, Nepal; ^4^Organization for Educational Initiatives, University of TsukubaTsukuba, Japan; ^5^Department of Anatomy I, Showa University School of MedicineShinagawa, Japan

**Keywords:** glutathione, oxidative stress, redox sensing, gamma-glutamyltransferase, plant acclimation

## Apoplast and redox components in plants acclimation to environment

The apoplast represents a compartment where an extensive cross-talk occurs among different components, to generate signals that can pass through the plasmalemma and reach the symplast (Agrawal et al., 2010). Both abiotic and biotic stress conditions evoke defensive and adaptive responses. Occurrence of structural and metabolic readjustments is then driven by enzymes (proteins), whose coordinated expressions are regulated by signals and signal transduction pathways (Foyer and Noctor, 2005).

External environmental factors initiate extracellular signals. Signals are then transferred to inner compartment via receptors located on the plasma membrane initiating a signal transduction pathway to readjust cell metabolism to the new conditions. This task requires a concerted action of many players: specific genes expression, post-transcriptional and post-translational regulation, hormones, and cell regulators.

When trying to explain the process of plant sensing and acclimation to environment, key questions arise: what are the signals generated by the environment? How can they evoke the response? A widely accepted view is that many unfavorable conditions result in the appearance of reactive oxygen species (ROS) (Pitzschke et al., 2006). ROS are the natural consequence of a life in an oxygen-containing atmosphere, and result from any imbalance in the electron flow in fundamental processes such as photosynthesis and respiration. They are represented by oxygen-containing radical species or hydrogen peroxide, H_2_O_2_, having an intrinsic reactivity with the organic molecules which can be consequently either damaged or undergo a redox modification. The ROS increase in apoplast under oxidative conditions has been documented (Mittler et al., 2004; Potters et al., 2010). ROS are involved in cell wall synthesis, remodeling and plant-pathogen interactions (Torres et al., 2006). ROS and redox modifications thus seem to be good candidates in transferring the environment-related information to cell, together with other signaling molecules, such as the extracellular ATP (Cao et al., 2014). Integration of apoplastic and chloroplastic ROS signaling processes has also been studied in different model organisms during stress conditions, suggesting that the extracellular ROS signal is transduced to chloroplasts, thus initiating a secondary and amplified ROS production (reviewed in Shapiguzov et al., 2012).

## Gamma-glutamyl cycle and gamma-glutamyl-transferases

In plants, the gamma-glutamyl cycle is a metabolic route of extra-cytosolic (apoplastic and vacuolar) glutathione degradation by gamma-glutamyl-transferase (GGT) and cys-gly dipeptidase, followed by the re-uptake of constituent amino acids, intracellular re-synthesis and extrusion (Ferretti et al., 2009). Ohkama-Ohtsu et al. (2008) demonstrated that an alternative pathway of glutathione degradation by means of gamma-glutamyl cyclotransferase (GGCT) and 5-oxo-prolinase (5Opase) dominates over GGT degradation in plant tissues. However, the two pathways operate in different compartments; GGTs are extracytosolic (apoplastic and vacuolar) whereas GGCT and 5OPase activities are restricted in the cytosol. The two degradation pathways coexist and operate independently of one another, and have therefore distinct physiological significance and regulation. Thus, the gamma-glutamyl cycle involving apoplastic GGTs is functional to the recovery of extracellular glutathione, whereas the alternative GGCT/5OPase pathway participates in controlling cytosolic glutathione homeostasis (Noctor et al., 2011).

In Arabidopsis, a detailed description of the four GGT genes expression was obtained by GUS-staining of transformed lines (Martin et al., 2007). GGT1 and GGT2 have high similarity and sequence identity, and are located to the apoplast (Ferretti et al., 2009). The GGT1 is ionically cell-wall bound and expressed in most vascular tissues (Ferretti et al., 2009), whereas GGT2 seems to be preferentially associated to plasma membranes and expressed in specific tissues in seeds, flowers, and roots. GGT3 is considered a non-functional and truncated sequence, whereas GGT4 is localized to vacuole assisting degradation of the GS-conjugates of toxic compounds and xenobiotics (Grzam et al., 2007). The significance of GSH cycling between the extracellular and intracellular space was addressed in the Arabidopsis mutant line lacking the *ggt1* isoform by performing comparative quantitative proteomics of the total leaf proteins (Tolin et al., 2013). In that study, it was reported that disrupture of the gamma-glutamyl cycle by *ggt1* silencing results in increased abundance of an array of antioxidant and defense protein enzymes, which could be collectively described as a “constitutive alert response.”

The occurrence of glutathione in apoplast has often been questioned in the past, but several evidences now indicate its existence albeit at low level (Zechmann, 2014). It seems however puzzling that a glutathione degradation activity, occurring outside the cell, can result in redox alteration inside the cell. Due to its low extracellular concentration, it is unlikely that glutathione itself acts as an antioxidant outside the cell. That function might better be fulfilled by abundant ascorbate in apoplast (Pignocchi and Foyer, 2003); where in any case oxidizing conditions are prevalent.

## Extracellular glutathione and glutathione degradation activity

All this considered, what could be then the function of extracellular glutathione and glutathione degradation activity? Some key elements worth considering are: (i) presence of a redox-sensitive thiol group in the molecule; (ii) apoplastic ROS production as a consequence of adverse conditions; (iii) presence of the plasma-membrane bound receptors; and (iv) redox exchange reactions occurring between the low-molecular-weight thiols and cysteines of plasma-membrane bound proteins, acting as redox switches. In order for a molecule to act as a signal, its concentration should be low and un-buffered, such that perturbations may induce large variations in its pool size. The reversible conversion of reduced to oxidized form may also rapidly modify the GSH pool. The interaction and exchange reactions of low-molecular-weight thiols and cysteines of plasma-membrane receptors and components may secondarily amplify the signal. On the other hand, the possibility that gamma-glutamyl cycling be implicated in the response to oxidative stress might be inferred by some previous reports (Masi et al., 2002; Ferretti et al., 2009).

To better investigate the relationship between oxidizing stress conditions and GGT-driven glutathione degradation, apoplastic fluid proteins were extracted from leaves of the *ggt1* mutant following ultraviolet B (UV-B) treatment (Trentin et al., 2015). Comparative quantitative proteomics suggests that while abundance of cell wall remodeling proteins is affected by both UV-B and *ggt1* silencing, the mutation itself resulted in reduced expression of a number of plasma-membrane associated genes (cys-rich, leucine-rich secretory proteins) involved in signaling and assigned to “response to stimulus” as per the gene ontology. Alteration in expression of ROS components (i.e., superoxide dismutase, glutathione S-transferases or peroxidases) is also observed under stress conditions. But given the presence of parallel alternative pathways, it is hard to predict whether: (i) the level of apoplastic H_2_O_2_ is increased or not; and (ii) H_2_O_2_ is the molecule involved in transferring the signals arising from apoplast.

## Proteomics as a tool to understand the gamma-glutamyl cycle

Proteomics technology has been very useful in better understanding the gamma-glutamyl cycle by using the GGT mutant plants. It was a proteomics study of the *ggt1* mutants that provided evidence on alterations in abundance of protein components involved in antioxidative and defense responses, and that may convey redox information from the extracellular milieu to internal compartments. In the future, proteomics may contribute to pinpoint plasma membrane components that are clearly involved in this process. Proteomics may also help in identifying one missing step in the gamma-glutamyl cycle, i.e., the cysteinyl-glycine dipeptidase, whose occurrence is inferred but not demonstrated yet.

## Conclusions

**Figure 1:**
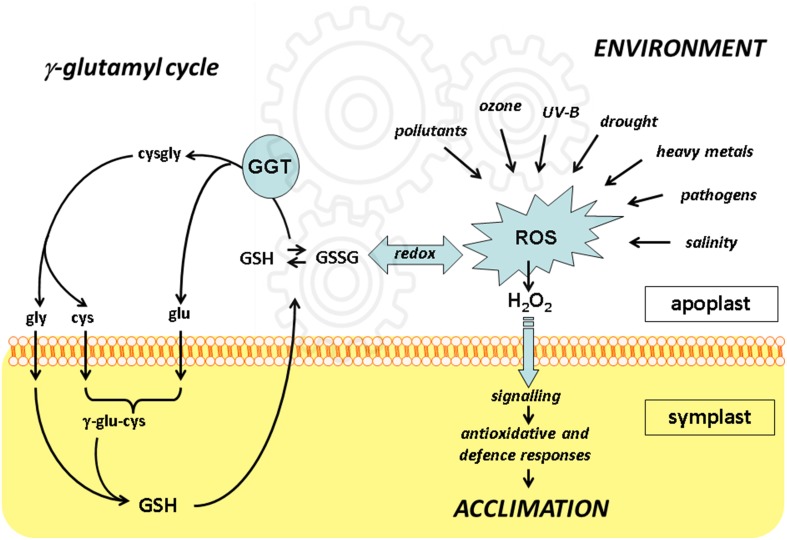
**The integration of redox events in apoplast with the γ-glutamyl cycle**. Unfavorable environmental conditions result in formation of reactive oxygen species (ROS) including hydrogen peroxide (H_2_O_2_), which may intracellularly activate anti-oxidative and defense responses leading to plant acclimation.

The significance of the gamma-glutamyl cycle is not fully understood yet. Glutathione cycling between the symplast and apoplast may represent a way to transfer redox information. Functional genomics approaches indicate that disruption of the functional cell-wall bound GGT1 isoform results in a constitutive alert response where anti-oxidative enzymes are up-regulated, probably as an effect of the altered plasma membrane receptors level and the redox state. With the more general aim of understanding how environmental challenges are perceived by plant cells, it seems therefore important to conclusively assign a role for extracellular GGTs and the gamma-glutamyl cycle in controlling the redox signals generated in apoplast. To this end, further high-throughput and targeted proteomic approaches will be necessary to perform and compare under the diverse stresses as indicated in Figure 1.

### Conflict of interest statement

The authors declare that the research was conducted in the absence of any commercial or financial relationships that could be construed as a potential conflict of interest.
